# Active head movements contribute to spatial updating across gaze shifts

**DOI:** 10.1098/rsos.231545

**Published:** 2024-08-07

**Authors:** Manuel Bayer, Eckart Zimmermann

**Affiliations:** ^1^ Department of Experimental Psychology, Heinrich-Heine-University Düsseldorf, Düsseldorf 40225, Germany

**Keywords:** space constancy, self-motion, head movement, eye movement

## Abstract

Keeping visual space constant across movements of the eye and head is a not yet fully understood feature of perception. To understand the mechanisms that update the internal coordinates of space, research has mostly focused on eye movements. However, in natural vision, head movements are an integral part of gaze shifts that enlarge the field of vision. Here, we directly compared spatial updating for eye and head movements. In a virtual reality environment, participants had to localize the position of a stimulus across the execution of a gaze shift. We found that performing head movements increased the accuracy of spatial localization. By manipulating the speed of the visual scene displacement that a head movement produced, we found that spatial updating takes into account the sensorimotor contingencies of vision. When we presented gaze-contingent visual motion, subjects overestimated the position of stimuli presented across gaze shifts. The overestimation decreased if subjects were allowed to perform eye movements during the head movement. We conclude that head movements contribute to stabilizing visual space across gaze shifts and that contingencies of head movements, rather than being cancelled, facilitate the updating.

## 1. Introduction

Obtaining information quickly and efficiently is crucial for our interaction with the environment. The sensorimotor system displaces the eyes with a frequency of about 3 Hz at high speed in order to enable us to rapidly access information in our environment. These eye movements, called saccades, bring the point of highest resolution, the fovea, onto regions of interest. Gaze shifts come at a cost: the sensorimotor system must distinguish between motion on the retina, produced by the saccade or the head movement and motion occurring in the external world [1,2]. To this end, the system must know about the amplitude of the performed movements. Electrophysiological studies found that neurons in the lateral intraparietal area [3] and visual area V3 [4], about 50 ms before the eye starts moving, receive information from spatial locations that would fall into the neuron’s receptive field once the saccade has landed. This process has been termed remapping and is likely responsible for creating a transient supra-retinal reference frame at least for a few attended items [5–9].

Several behavioural experimental setups have been established to measure the integration of vision across the execution of saccades in the laboratory [9–11]. In a convenient method, observers are asked to compare the position of a stimulus presented before the saccade performance against the location of a second stimulus presented after the saccade has been terminated [9]. Localization across gaze shifts has mostly been studied in saccades, while the head was immobile. However, in larger gaze shifts, saccades are accompanied by head movements that expand the field of vision and align our visual field with the region of interest. Head movements start contributing to the gaze shift when targets are presented at an eccentricity larger than 20° [12]. Studies that tested localization across eye-head gaze shifts found evidence for an accurate updating process [13,14].

In principle, internal knowledge about the saccade vector might be sufficient to bridge the pre- and post-saccadic visual space. However, head movements could contribute to spatial localization across gaze shifts by potentially adding three signals to the remapping process. Rotational head movement signals are detected by the semicircular canals and processed initially in vestibular neurons in the cerebellum and brainstem [15]. Active and passive head movements are distinguished by the response strength of the vestibular neurons. During active movements, activation of these neurons is attenuated [16]. In addition to vestibular signals, neck proprioception information modulates the responses of vestibular neurons. However, this effect cannot be generated by passive head movements. Only if the intended head movement matches the actual head movement does the vestibular processing become attenuated. These findings suggest the existence of an internal model that predicts the sensory consequences of an upcoming movement and suppresses the actual sensorimotor contingencies if they match the expectation.

Head movements last on average between 400 and 800 ms [17,18]. During head movements, eye movements, including saccades and eye movements that are triggered by the vestibulo-ocular reflex and the optokinetic nystagmus, are performed [19,20]. The vestibulo-ocular reflex causes eye movements in the opposite direction of the performed head movement in order to keep the gaze aligned with the orientation of the head. The optokinetic nystagmus on the other hand is a sawtooth movement of the eye, which aims to stabilize a moving image on the retina. The involvement of the head movement in the context of gaze shifts and localization is also highlighted by the fact that gaze shifts including both types of movement are initially coded as an integrated signal and are only later—downstream of the superior colliculus—separated into individual signals [12,21,22].

In the present study, we asked how head movements affect spatial updating across gaze shifts. We measured spatial updating by asking subjects to localize a target seen during the fixation of the eye and the head after the execution of an eye-head gaze shift. The accuracy and precision of spatial localization and the amplitude of the eye and head gaze shift components revealed to what extent human observers can compensate for the displacement of the eyes and the head. We used a head-mounted display to present visual stimuli and to simultaneously record head and eye movements. In experiments 1 and 2, we varied the availability of visual references and visual backgrounds across conditions. We manipulated the availability of visual references, by either presenting the gaze shift targets continuously or by briefly flashing them. Saccade targets that are presented only shortly elicit a stronger saccade undershoot [23,24]. We wondered if head movements would similarly undershoot the targets or if they could even compensate for the undershoot.

In experiment 3, we manipulated the background motion by either using a static background or rotating the background against the direction of the performed eye-head movement. Since most head movements last long enough that visual information is analysed during the movement [18], visual background information might influence updating across an eye-head gaze shift. This allowed us to explore the use of background motion as a source of information in the context of spatial remapping processes.

## 2. Methods

### 2.1. Participants

The sample of experiments 1 and 2 consisted of 35 participants. Owing to poor task performance, 11 participants had to be excluded from the analyses. The final sample included 18 females and 6 males with a mean age of 24.29 years (24.29 years ± 0.84 years). The sample of experiment 3 consisted of 37 participants. Owing to poor task performance, 11 participants had to be excluded from the analyses. The final sample included 24 females and 2 males with a mean age of 21.04 years (21.04 years ± 0.41 years). Owing to the within-subject design applied in the analyses, it was necessary to exclude every participant that did not meet the inclusion criteria in a single sub-condition of the experiments the respective participant participated in. In order to be included, the participants had to be able to perceive the stimulus of interest within the presented stimuli range. Failing to fulfil this criterion indicated that the respective participant did not perceive the stimuli accurately enough to draw meaningful comparisons. Every participant gave written informed consent prior to the experiment in accordance with the declaration of Helsinki, participated voluntarily and received either course credit or monetary compensation. This study was approved by the local ethics committee of the mathematical and natural science faculty at Heinrich-Heine-University Düsseldorf (Ethics approval associated with ERC grant 757184).

### 2.2. Setup

Stimuli were presented by a custom program created with Unreal Engine (v. 4.26), running on a Windows 10 desktop computer (Alienware Aurora R8, Intel Core i7 8700 @3.2 GHz, 16 GB RAM, NVIDIA GeForce RTX 2080 graphics card). The used head-mounted display was an HTC Vive Pro with two dual AMOLED 3.5″ screens, a resolution of 1440 × 1600 pixels per eye (2880 × 1600 pixels combined), a refresh rate of 90 Hz and a field of view of 110°. The built-in 120 Hz eye tracker was used to record the eye position and a custom Python app, working at a mean sample rate of 913.68 Hz (913.68 Hz ± 0.74 Hz), to record the head position. The virtual environment was run using SteamVR (v. 1.25.3) with the SteamVR 2.0 tracking system. Previous research has shown that the system provides suitable tracking of head and hand positions for research purposes if tracking loss is prevented [25,26]. All positions of stimuli are given in rotational degrees.

### 2.3. Procedure

#### 2.3.1. Experiment 1: localization across eye-head gaze shifts

**Figure 1.**
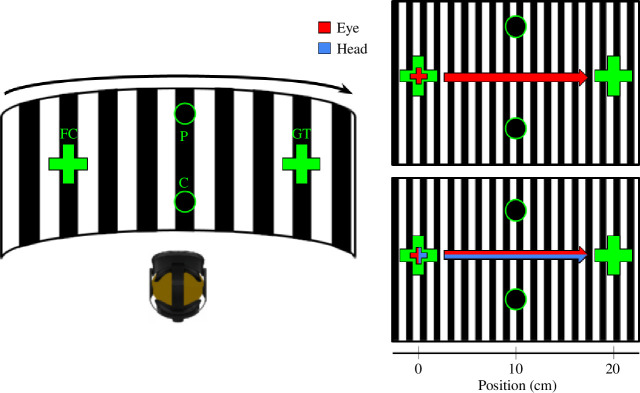
Illustration of the experimental setup. Participants performed either eye-only or eye-head gaze shifts from the fixation cross (FC) to the gaze shift target (GT). Participants had to judge the position of the comparison stimulus (C), presented after the gaze shift, against the probe stimulus (P), presented prior to the gaze shift. Please note, the displayed grating was only visible during the gaze shift and not during the presentation of the stimuli.

The first experiment was divided into eight sessions. Each session started with a short exploration of the virtual environment followed by 10 training trials and 56 experimental trials. The virtual environment of this experiment consisted of a grey background void of any form of reference. All stimuli presented during the experiment were shown 250 cm in depth from the participants’ point of view. Throughout each trial, two crosses (3.44° × 3.44°) were presented, at an eccentricity of 10° to the left and right of the centre view. The left cross was the fixation cross while the right was the gaze shift target. At the beginning of each trial, participants were instructed to fixate the fixation cross (shown in green colour) with their eyes and heads. Ocular fixation was counted as successful if the eye remained within an invisible circular window of 1° around the fixation cross. After the fixation cross was fixated successfully for 55 ms, a black dot-shaped probe stimulus with a radius of 10 cm was presented either 22° above or below the centre view direction for 400 ms. During this period, the participants were required to maintain fixation at the fixation cross. If they failed to maintain fixation on the fixation cross, the trial was aborted and restarted. After the stimulus disappeared, the fixation cross turned red and the gaze shift target turned green indicating that the participants should perform a gaze shift to the target and fixate it with their eyes and heads. The participants were explicitly instructed to perform a combined eye-head movement to the gaze shift target. After a successful fixation of the gaze shift target, a black dot-shaped reference stimulus, i.e. the visual comparison stimulus, was presented on the opposite vertical position of the probe, again either 22° below or above the centre view direction for 400 ms (see figure 1). During this period, the participants were required to fixate on the gaze shift target. The comparison stimulus could appear at seven different positions ranging from 1.37° to the left to 1.37° to the right of the centre view direction. The exact possible shift steps were −1.37°, −0.92°, −0.46°, 0°, 0.46°, 0.92° and 1.37°; negative values indicate a shift to the left. Each position of the comparison stimulus was presented eight times within a session, resulting in a total of 56 trials. At the end of a trial, the participants had to respond if they perceived the comparison stimulus to the left or right of the probe in a two-alternative forced choice task. The participants gave their response by pressing the touchpad of an HTC Vive Controller, which they were holding with their dominant hand.

In eight different experimental sessions, we varied the visual stimulation during the gaze shift. We either presented (i) a homogeneous grey background with a stationary fixation cross and gaze shift target, (ii) a grey background with a fixation cross and gaze shift target that were only briefly flashed, (iii) a background consisting of a whole-field grating and a stationary fixation cross and gaze shift target, or (iv) a whole-field grating and a flashed fixation cross and gaze shift target. These four visual stimulation conditions were presented under two different gaze shift instructions. The participants had to move their eyes and heads either in an unrestricted manner or sequentially. In the unrestricted condition, the participants could move their eyes and heads in a free, self-paced fashion. In the sequential condition, the participants were required to first perform a saccade to the gaze shift target, maintain ocular fixation and then move their heads. During the execution of the head movement, they had to keep ocular fixation on the gaze shift target.

#### 2.3.2. Experiment 2: localization across eye-only gaze shifts

In experiment 2, subjects were instructed to perform a saccade to the gaze shift target while keeping their heads immobile. For this purpose, a chin rest was used. We applied the same four manipulations of visual stimulation as in experiment 1.

#### 2.3.3. Experiment 3: background motion in eye-head gaze shifts

In experiment 3, the participants had to perform eye-head gaze shifts. The participants were either unrestricted in their performance of the gaze shift or had to start the gaze shift with an eye movement. After the performance of the eye movement, they had to fixate the gaze shift target with their eyes during the execution of the head movement. The fixation cross and gaze shift target were stationary, i.e. presented throughout the whole trial and a grating was presented during the movements from the fixation cross to the gaze shift target. The grating was moving against the direction of the head movement with three visual velocity gains, either as fast as the head movement (gain: 1) or faster (gains: 1.15, 1.3). The required gaze shift amplitude was 20°. For instance, when a visual velocity gain of 1.15 was active and a head movement of 20° was performed, the grating shifted 23° to the left, against the direction of the head movement. The order of all conditions within all three experiments was randomized for each participant and counterbalanced across participants. With three visual velocity gains and two gaze shift instructions (unrestricted, sequential), experiment 3 consisted of six conditions.

### 2.4. Head and eye movements

To analyse the head movement data, we first determined the start and end position of the individual movements. The data were analysed with regard to the rotation around the vertical axis (yaw). Moving averages were used to smoothen the data which took ten consecutively following visual velocity values into account.

Afterwards, the time point of the peak velocity of the movement was determined. The start and end of the movement were determined by checking when the visual velocity values were below 3°/s before and after the peak velocity time point. Trials in which the respective participant failed to exceed the visual velocity of 3°/s or performed a head movement smaller than 10° were excluded. If a participant performed a head movement of less than 10° in a trial, the head movement did not cover half of the distance between the fixation cross and the target, which is problematic for the comparisons, especially between eye and eye-head gaze shifts. These criteria led to the exclusion of 2.72% of trials across all participants.

**Figure 2.**
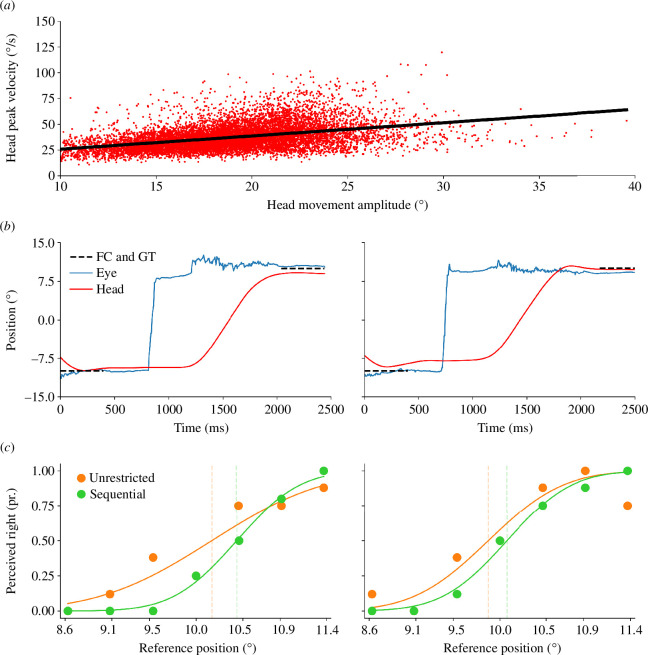
(*a*) Average head movement main sequence, illustrating the linear relationship (y = 13.22 + 1.27x) between head movement amplitude and peak velocity. Data were collapsed across trials and participants in the first experiment. (*b*) Gaze shift traces for the head movement (shown in red) and the eye movement component (shown in blue) from an example trial in which the participant performed an unrestricted (left) and sequential gaze shift (right). The dashed lines represent the position of the fixation cross and gaze shift target on the horizontal axis. The eye moves to the target first, followed by the rotation of the head. (*c*) Psychometric functions of two representative participants for the stationary target - grating condition split by the eye movement restriction during the head movement.

To check if the performed head movements were in line with common head movement dynamics reported in the literature [20,27], we analysed the amplitude in relation to the achieved peak velocity of the individual head movements in experiment 1 (see figure 2*a*). The participants moved their heads further to the sides when the achieved peak velocity was higher during the head movement (one-sample, paired *t*‐test; *t*(23) = 12.23, *p* < 0.001). The same holds true for the third experiment; the participants moved their heads further to the sides when the achieved peak velocity was higher during the head movement (one-sample, paired *t*‐test; *t*(25) = 13.90, *p* < 0.001).

To analyse the eye data, we determined all saccades performed within one respective trial. As the 120 Hz eye tracker did not allow the use of standard velocity-based detection algorithms, we used an algorithm that uses amplitude changes between individual data points irrespective of the time that has passed between the recording of the consecutive data points. We first calculated the differences between individual consecutive positional data points. Saccade onset was defined as the first data point prior to a difference of 1°. Saccade offset was defined as the data point recorded at least 30 ms later than the onset and deviated less than 0.1° from the previous data point. Based on visual inspection, this procedure yielded appropriate results with regard to the detection of individual saccades performed within a trial.

### 2.5. Statistical analysis

#### 2.5.1. Experiment 1

To investigate the differences between unrestricted and sequential gaze shifts in the first experiment, we analysed the fixation duration of the gaze shift target with the eye during the head movement component of the gaze shifts the participants performed. Fixation duration was quantified as a mere measure of how the participants moved their eyes during the head movement component. A low fixation duration implies that many eye movements were performed during the head movement component. We analysed the influence of the presentation of the targets, the presence of the grating and the performance of the gaze shift by performing a repeated measures ANOVA with the within-subject factors target presentation (stationary and flashed), presence of the grating (grating and no grating) and gaze shift performance (unrestricted and sequential). If sphericity was not given, the Greenhouse–Geisser correction was applied. To resolve significant interactions, we computed two-tailed Bonferroni corrected *t*-tests. We then performed the same ANOVA described above to analyse the amplitudes of the performed eye and head movements.

The next step in our analysis was to estimate the horizontal position at which the participants perceived the stimulus presented before and after the gaze shift to be aligned vertically. To this end, we calculated psychometric functions, based on the average responses indicating whether the comparison stimulus was to the left or to the right of the probe stimulus. The horizontal position where the cumulative Gaussian function reached 50% was chosen as the point of horizontal alignment (PHA). We calculated the PHA and the just-noticeable difference (JND) of every individual participant in each condition of the first experiment. To analyse the resulting PHA and JND values, we performed the same ANOVA described above with the respective values. The PHA represented the accuracy of the judgement of the participants, e.g. how close their judgement of the vertical alignment of the two stimuli was compared to their objective position. The JND on the other hand represented the precision or discrimination sensitivity, e.g. how well the participants were able to differentiate between different stimuli positions.

#### 2.5.2. Experiment 2

To analyse the eye movements performed in experiment 2, we performed a repeated measures ANOVA with the within-subject factors target presentation (stationary and flashed) and the presence of a grating (grating and no grating) on the saccade amplitudes of eye-only gaze shifts. To analyse the psychometric data of experiments 1 and 2 together, we performed a repeated measures ANOVA with the within-subject factors gaze shift (eye-only and eye-head), target presentation (stationary and flashed) and the presence of a grating (grating and no grating) for the PHA and JND.

#### 2.5.3. Experiment 3

To analyse the fixation durations observed in experiment 3, we performed a repeated measures ANOVA with the within-subject factors gaze shift (unrestricted and sequential) and visual velocity gain (unity visual velocity, 1.15, 1.3). The same repeated measures ANOVA was used to analyse the observed saccade and head movement amplitudes.

To test if the performance of the gaze shift had an effect on the localization accuracy we performed regression analyses. We fitted linear functions to the visual velocity gain values and their respective PHAs on the single subject level. For each participant, we determined the PHA for unity visual velocity gain for a visual velocity gain of 1.15 and 1.3. The resulting three values were used to fit a linear function with a least squares procedure. This procedure was performed separately for unrestricted and sequential gaze shifts. Additionally, we compared the slopes of the unrestricted and restricted movement condition against 0 in order to test how the application of visual velocity gains influenced localization accuracy. If the introduction of higher visual velocity gains did not influence the accuracy of the participants’ judgement, the slope values should on average not deviate from 0. We performed the same regression analysis for the localization sensitivity.

## 3. Results

### 3.1. Experiment 1: localization across eye-head gaze shifts

#### 3.1.1. Eye and head movements

**Figure 3.**
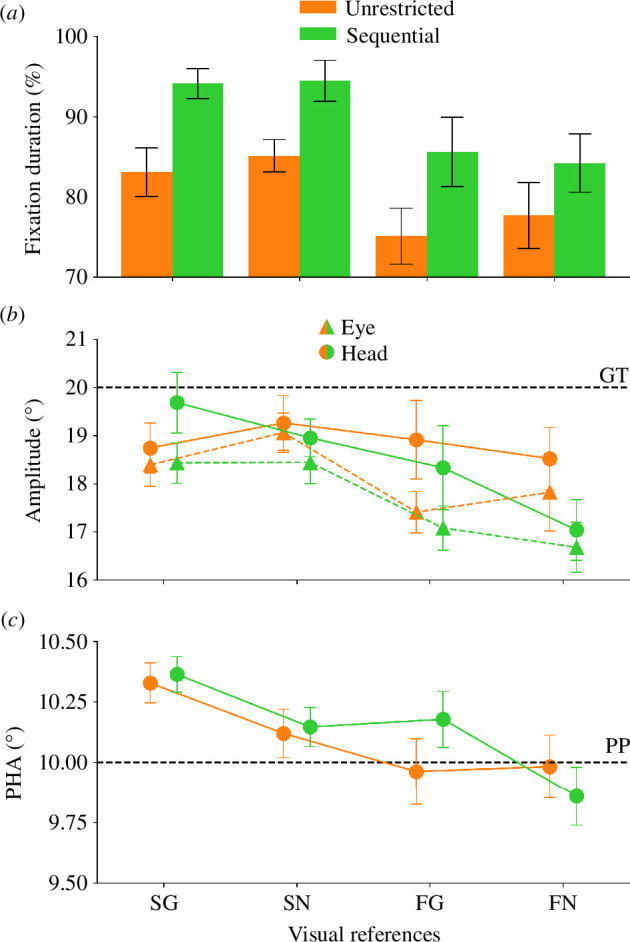
(*a*) Duration of the fixation of the gaze shift target during the execution of the head movement component of the gaze shift for the stationary target - grating (SG), stationary target - grey background (SN), flashed - grating (FG) and flashed - grey background condition (FN) split by the eye movement restriction during the head movement. (*b*) Saccade and head movement amplitudes for the individual conditions. The black dashed line represents the position of the gaze shift target (GT). Triangles connected by colored dashed lines represent the amplitudes of the eye movements, whereas circles with solid lines represent the amplitudes of head movements. Same conventions as in (*a*). (*c*) PHAs for the individual conditions. The dashed line represents the position of the probe (PP). Same conventions as in (*a*).

In experiment 1, the participants had to move their eyes and heads either in an unrestricted or in a sequential manner and had to localize the remembered position of a stimulus, seen before the gaze shift. We first analysed head and eye movement parameters. The participants performed saccades with a mean amplitude of 17.97° (17.97° ± 0.38°). Head movement dynamics followed the main sequence [20] with an average peak velocity of 37.01°/s (37.01°/s ± 1.83°/s), a mean amplitude of 18.83° (18.83° ± 0.46°) and a duration of 765.40 ms (765.40 ms ± 28.55 ms) on average (see figure 2*a*). Figure 2*b* illustrates the sequence of movements performed during these gaze shifts. One can see that the participants first performed a saccade and then a head movement to the gaze target. Figure 3*a* displays the eye fixation durations for the different conditions split by the type of gaze shift.

The participants fixated the gaze target for most of the duration of the head movement (>80% across all conditions). There was a significant main effect of the factor target presentation on the eye fixation duration (*F*_1,23_ = 18.49, *p* < 0.001, *η* ^2^ = 0.45, power = 0.98), stationary targets led to longer fixations (89.23% ± 1.31%) compared with flashed targets (80.68% ± 2.01%). The performance of sequential gaze shifts also increased the fixation duration (89.64% ± 1.68%) compared with unrestricted gaze shifts (80.28% ± 1.69%; *F*_1,23_ = 48.54, *p* < 0.001, *η* ^2^ = 0.68, power = 0.99).

There were no other significant effects (all *p* ≥ 0.471). We next checked for differences in saccade and head movement amplitudes in relation to the available visual references. Figure 3*b* displays the eye movement (triangle) and head movement amplitudes (circle) for the unrestricted (orange) and sequential gaze shifts (green). One can see that both eye and head movements had roughly the same amplitudes across conditions and that both movements generally undershot the gaze target. Descriptively, this undershoot became larger when fewer visual references were available.

There was a significant main effect of the factor target presentation for the eye movement amplitudes (*F*_1,23_ = 26.51, *p *< 0.001, *η* ^2^ = 0.54, power = 0.99). The participants undershot more when the targets were briefly flashed (17.25° ± 0.29°) compared with when they were stationary (18.58° ± 0.22°). The same could be observed for head movements; there was a significant main effect of the factor target presentation on the head movement amplitude (*F*_1,23_ = 5.59, *p* = 0.027, *η* ^2^ = 0.20, power = 0.62), the participants undershot more when the targets were flashed (18.20° ± 0.38°) compared with when they were stationary (19.16° ± 0.27°). There was also a significant interaction between the performance of the gaze shift and the target presentation for head movement amplitudes (*F*_1,23_ = 6.88, *p* = 0.015, *η* ^2^ = 0.23, power = 0.71). The participants undershot less in unrestricted gaze shifts when the target was stationary (19.31° ± 0.38°) compared with when the target was flashed (17.69° ± 0.55°; *t*(23) = 3.12, *p* = 0.005). There were no other significant effects for saccade and head movement amplitudes in experiment 1 (all *p *≥ 0.085).

#### 3.1.2. Psychometric data

The PHA and the JND of two representative participants for each condition of the first experiment can be seen in figure 2*c*. Figure 3*c* illustrates the mean PHAs for the individual conditions. We descriptively observed more overcompensation when more visual references were available to the participants, i.e. the participants shifted the position of the probe stimulus further of the gaze shift than actually required. There was a significant main effect of the factor target presentation on the PHA (*F*_1,23_ = 11.00, *p* = 0.003, *η* ^2^ = 0.32, power = 0.89). The participants overcompensated more for stationary (0.24° ± 0.04°) compared with flashed targets (0.00° ± 0.06°). There was also a main effect of the factor grating presence on the PHA (*F*_1,23_ = 12.30, *p* = 0.002, *η* ^2^ = 0.35, power = 0.92). The presence of a grating as a background also led to more overcompensation (0.21° ± 0.06°) compared with a grey background (0.03° ± 0.06°). There were no other significant results regarding the PHA (all *p* ≥ 0.161).

The ANOVA performed for the JNDs revealed a main effect of the factor target presentation (*F*_1,23_ = 9.68, *p* = 0.005, *η* ^2^ = 0.30, power = 0.85). The participants were more sensitive to positional differences when the targets were presented stationary (0.76° ± 0.03°) compared with when they were flashed (0.96° ± 0.04°). There were no other significant results (all *p ≥ *0.325).

### 3.2. Experiment 2: localization across eye-only gaze shifts

#### 3.2.1. Eye movements

In experiment 2, we let the participants perform only saccades with a fixed head position. The participants performed saccades with a mean amplitude of 16.78° (16.78° ± 0.32°). The mean head position was at 1.28° (1.28° ± 0.48°) and differed significantly from a rotation of 0, *t*(23) = 2.61, *p* = 0.015. No head movements were detected across all trials performed.

**Figure 4.**
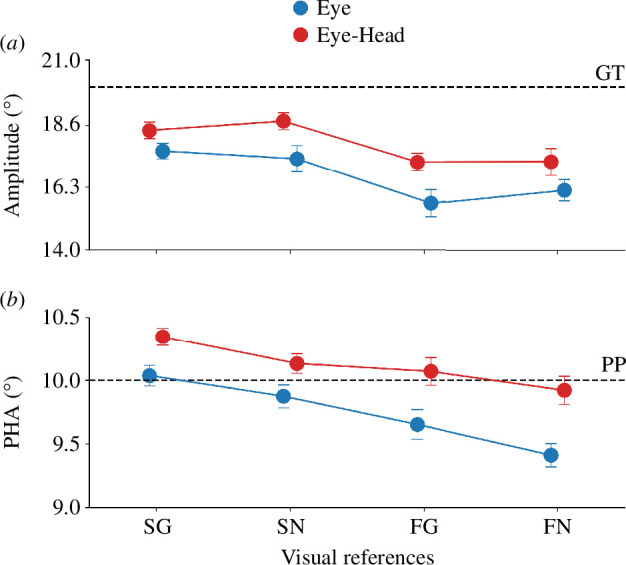
(*a*) Saccade amplitudes for the stationary target - grating (SG), stationary target - grey background (SN), flashed - grating (FG) and flashed - grey background condition (FN) of the first and second experiment split by color. The dashed line represents the position of the gaze shift target (GT). (*b*) PHAs for the individual conditions of the first and second experiment. The dashed line represents the position of the probe (PP). Same conventions as in (*a*).

Figure 4*a* illustrates the amplitudes of eye-only (blue, experiment 2) and eye-head gaze shifts (red, experiment 1). One can see that the participants undershot the gaze target in both types of gaze shift and that this undershoot was more pronounced in conditions with fewer visual references. There was a significant main effect of the factor target presentation on the saccade amplitudes of eye-only gaze shifts (*F*_1,23_ = 14.33, *p* = 0.001, *η* ^2^ = 0.38, power = 0.99). The participants undershot more when the targets were flashed (15.96° ± 0.31°) compared with when they were stationary (17.51° ± 0.29°). There were no other significant results (all *p* ≥ 0.154).

#### 3.2.2. Psychometric data

Next, we wanted to compare the localization accuracy between eye-head and eye-only gaze shifts. Figure 4*b* shows the PHAs for eye-only (experiment 2) and unrestricted eye-head gaze shifts (experiment 1) for the individual conditions. Descriptively, for both eye-only and unrestricted eye-head gaze shifts, the compensation accuracy decreased with fewer visual references. Eye-only gaze shifts led overall to more undercompensation, while unrestricted eye-head gaze shifts tended to lead to overcompensation. There was a main effect of the factor gaze shift on the PHA (*F*_1,23_ = 34.73, *p *< 0.001, *η* ^2^ = 0.60, power = 0.99). The participants undercompensated more when they performed eye-only (−0.26° ± 0.05°) compared with eye-head gaze shifts (0.10° ± 0.06°). There was also a main effect of the factor target presentation on the PHA (*F*_1,23_ = 16.60, *p *< 0.001, *η* ^2^ = 0.42, power = 0.99). Flashed targets also led to more undercompensation (−0.25° ± 0.06°) compared with when they were stationary (0.09° ± 0.05°). The main effect for the factor presence of a grating was also significant for the PHA (*F*_1,23_ = 5.09, *p* = .023, *η* ^2^ = 0.20, power = 0.64). When a grey background was presented during the gaze shifts, the participants also undercompensated more (−0.15° ± 0.06°) compared with when the background was a grating (0.00° ± 0.06°). There were no other significant effects (all *p ≥ *0.145).

There was a significant main effect of the factor target presentation on the JND (*F*_1,23_ = 8.85, *p* = 0.007, *η* ^2^ = 0.28, power = 0.81). The participants were more precise in their spatial judgement when the targets were stationary (0.70° ± 0.03°) compared with when they were flashed (0.90° ± 0.04°). There were no other significant differences (all *p *≥ 0.088).

### 3.3. Experiment 3: background motion in eye-head gaze shifts

#### 3.3.1. Eye and head movements

We then asked whether the sensorimotor system might take into account the visual motion that is contingently produced by a head movement. To this end, we artificially moved the background during the execution of head movements. In experiment 3, the participants performed saccades with a mean amplitude of 17.38° (17.38° ± 0.26°). Head movement dynamics followed the main sequence for head movements [20] with an average peak velocity of 48.14°/s (48.14°/s ± 2.63°/s), a mean amplitude of 20.25° (20.25° ± 0.32°) and a duration of 940.73 ms (940.73 s ± 44.63 ms) on average.

**Figure 5.**
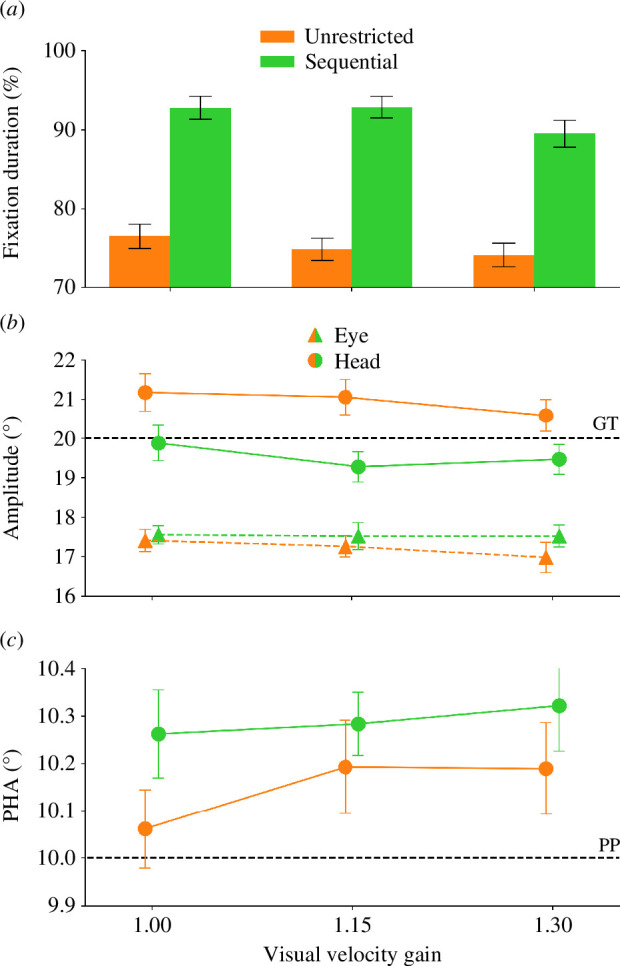
(*a*) Duration of the fixation of the gaze shift target during the execution of the head movement component of the gaze shift for the individual visual velocity gains split by the eye movement restriction during the head movement. (*b*) Saccade and head movement amplitudes for the individual visual velocity gains. The dashed line represents the position of the gaze shift target (GT). Same conventions as in (*a*). (*c*) PHAs for the individual visual velocity gains. The dashed line represents the position of the probe (PP). Same conventions as in (*a*).

First, we analysed the duration, the participants fixated the gaze shift target during their head movement. Figure 5*a* displays the fixation duration for the two types of gaze shifts and the different visual velocity gains. Also, in this experiment, the participants fixated the gaze target for most of the duration of the head movement. In sequential eye-head gaze shifts, the participants almost fixated the gaze target for the whole head movement duration.

There was a significant main effect of the factor gaze shift on the eye fixation duration (*F*_1,25_ = 73.76, *p* < 0.001, *η* ^2^ = 0.75, power = 0.99). The participants fixated the gaze shift target longer during their head movement in sequential (91.72% ± 0.89%) compared with unrestricted gaze shifts (75.12% ± 0.88%). The application of a visual velocity gain also had an impact on the fixation duration (*F*_2,50_ = 5.19, *p* = 0.009, *η* ^2^ = 0.17, power = 0.95). Bonferroni corrected post hoc dependent *t*-tests were able to reveal a significant difference between the unity visual velocity gain and a visual velocity gain of 1.3 (*t*(25) = 2.88, *p* = 0.024). The participants fixated the right fixation cross during the head movement for a longer duration while the unity visual velocity gain was active (84.63% ± 1.57%) compared with a visual velocity gain of 1.3 (81.83% ± 1.56%). There were no other significant differences regarding the fixation duration (all *p* ≥ 0.080).

Figure 5*b* shows the saccade and head movement amplitudes the participants performed for the two types of gaze shift and the different visual velocity gains. We observed less undershooting in head movements compared with saccades. Unrestricted gaze shifts produced the least undershooting across all movements. Little to no differences were observed for the undershoot in relation to the applied visual velocity gains. There were no significant undershoot differences between the performed saccades across conditions (all *p* ≥ 0.061). For head movements, there was a significant main effect of the factor gaze shift (*F*_1,25_ = 13.02, *p* = 0.001, *η* ^2^ = 0.34, power = 0.99).

The participants undershot less in unrestricted (20.93° ± 0.26°) compared with sequential gaze shifts (19.53° ± 0.23°; see figure 5*b*). There were no other significant effects for the performed head movements (all *p* ≥ 0.216).

#### 3.3.2. Psychometric data

Figure 5*c* illustrates the mean PHAs for the two types of gaze shifts and the individual conditions. One can see that for both gaze shifts and all visual velocity gains, the participants overcompensated for their movements. This overcompensation was even more pronounced in sequential eye-head gaze shifts. The participants overcompensated more when they performed sequential (0.26° ± 0.08°) compared with unrestricted gaze shifts (0.08° ± 0.08°; paired *t*‐test; *t*(24) = −2.90, *p* = 0.008).

The *t*-tests against 0 performed with the slopes of the unrestricted and restricted movement conditions revealed that the localization accuracy of the participants was influenced by the applied visual velocity gain when they performed unrestricted gaze shifts (*t*(24) = 2.03, *p* = 0.027) but not when they performed sequential gaze shifts (*t*(24) = 0.63, *p* = 0.268). The same regression analysis for the localization sensitivity did not reveal any significant differences (all *p ≥ *0.203).

Since we found a difference in localization accuracy between unrestricted and sequential gaze shifts in experiment 3 but not in experiment 1, we calculated the difference in the fixation duration of the saccade target during the head movement between unrestricted and sequential gaze shifts for each participant for both experiments. There was a bigger difference in the fixation duration during the head movement between unrestricted and sequential gaze shifts in experiment 3 (16.60% ± 1.90) compared with experiment 1 (9.36% ± 1.32%; unpaired *t*‐test; *t*(24) = −3.02, *p* < 0.001).

## 4. Discussion

In this study, we found that performing head movements contributes to the accuracy in spatial localization across gaze shifts. In natural vision, head movements are an integral part of gaze shifts. On the one hand, the head-movement component complicates the updating of visual space across gaze shifts. In an eye-head gaze shift two reference frames, that of the eye and that of the head, move and thus must be compensated for. On the other hand, the position of the head can be used by the sensorimotor system to measure the size of the gaze shift. Four signals provide information about the amplitude of a head movement and can contribute to spatial updating. Three of these signals are exclusively internal, i.e. changes in vestibular and proprioceptive states and the efference copy, which is a copy of the motor command that drives the head movement. The fourth signal consists of the self-produced visual motion on the retina that originates from the relative displacement between the head movement and the external visual scene.

Vestibular signals inform about head position extremely fast with a latency of only 14 ms [28]. Vestibular and neck proprioception inputs are probably combined to decode head position, as the convergence of both signals is required for posture, balance and vestibular spinal reflexes [29–31]. Like for saccades, an efference copy of the head movement amplitude could also be involved in the spatial localization of eye-head gaze shifts. The efference copy is likely involved in the build-up of an internal model predicting the sensory consequences, i.e. vestibular and proprioceptive changes, following the performance of head movements. The interaction of these three signals could provide predictively available, precise information on the head movement size. Combining this information with the internal estimate of the upcoming saccade vector might improve the accuracy of spatial updating across a gaze shift. In addition, even the visual motion that is produced by the head movement entails information about the head movement amplitude. The sensed visual motion velocity could be read out to determine the actual head-movement speed, allowing deviations from the predicted head-movement amplitude to be detected. Instead of suppressing this latter signal, as suggested by traditional theories [32,33], the sensorimotor system uses the information to monitor the spatial extent of the head movement.

We first directly compared spatial updating in eye and in combined eye-head gaze shifts. When the participants performed saccades without moving their heads, localization was accurate when a background was shown and the fixation and the saccade target were permanently visible. When we decreased the availability of visual references by showing no background or in addition by only flashing the fixation cross and the saccade target in the pure saccade condition, we found that the participants underestimated the location of the pre-saccadic stimulus. They undershot the target with their saccades and they also indicated the position of the localization stimulus closer to the fixation point. Previous findings have demonstrated that uncertain target information leads to the saccadic undershoot [34]. Similarly, visual localization also drifts towards the fovea when visual references are absent [35]. From the current results, we cannot determine whether subjects visually underestimated the target location because they undershot the target with their saccades or if their saccades went too short because they saw the target closer to the fovea. However, we found that when subjects performed combined eye-head gaze shifts their accuracy in visual target localization increased. Subjects localized the target close to its veridical position when performing a combined eye-head movement. Except in the stationary target - grating (see figure notes) condition, subjects localized the target close to veridical. In order to understand why subjects visually overestimated the target location in the SG condition, we performed a third experiment.

In experiment 3, we measured spatial localization across an eye-head gaze shift when the background either was stationary or when it moved against the direction of the head movement. The latter condition served to increase the experience of background motion. We compared localization performance when observers could freely execute an eye-head gaze shift or when they were required to keep their eye direction fixated on the target cross while executing the head movement. We found an overestimation of the target as a function of background displacement velocity in both conditions. However, the overestimation was significantly stronger when subjects were required to keep their eye direction fixated on the target cross. A moving grating induces an optokinetic nystagmus if observers do not maintain fixation. The nystagmus stabilizes the grating on the retina and thus partly cancels out the motion experience. In the unrestricted condition, when the background moved with unity gain, the participants localized the probe accurately. However, when the grating moved faster than the unity gain, subjects overestimated the probe position. The overestimation increased in the sequential condition when subjects were not allowed to perform eye movements. Without eye movements, the motion on the retina increases, since the optokinetic nystagmus cannot cancel out the motion. The motion on the retina is thus the most likely explanation for the overestimation of the probe position. Please note that the participants in our study had to wear a head-mounted display, which was light, but could have affected the information provided by the neck proprioception. However, this influence was identical throughout all conditions and should thus not affect our results systematically. Neither did head-movement amplitudes show any modulation by background motion nor was their distribution suited to explain where subjects localized the probe stimulus.

In a previous study, we showed that subjects are sensitive to deviations between the expected and the actual head movement—contingent visual motion velocity [36]. We found that even after a single experience of such a deviation, subjects shifted their expectation about the motion velocity occurring during head-movement execution. The need to maintain the accurate expectations about movement-contingent motion could lie in their contribution to spatial updating. Head movements arise in a gaze shift when desired objects have an eccentricity of 20° or more [12]. Targets at that distance will necessarily provide an uncertain visual signal as they fall on peripheral retinal locations. We found in the present study that uncertain visual signals generate saccades that undershoot their targets combined with an undercompensation of the visual space. A gaze shift consisting of an eye and a head movement component solves the problem since first, the combined gaze movement is more accurate and second, the visual motion produced by the head movement is used by the sensorimotor system to update the visual space. In order to isolate the motion on the retina from efference copy and predicted vestibular signals or neck proprioception, future studies could compare active and passive head movements. We conclude that the self-produced movement-contingent visual motion during a head rotation contributes to updating the visual space across gaze shifts.

## Data Availability

Data and code are available online [37].
